# Uncovering the Association Between m^5^C Regulator-Mediated Methylation Modification Patterns and Tumour Microenvironment Infiltration Characteristics in Hepatocellular Carcinoma

**DOI:** 10.3389/fcell.2021.727935

**Published:** 2021-09-13

**Authors:** Xinyu Gu, Haibo Zhou, Qingfei Chu, Qiuxian Zheng, Jing Wang, Haihong Zhu

**Affiliations:** State Key Laboratory for Diagnosis and Treatment of Infectious Diseases, National Clinical Research Center for Infectious Diseases, Collaborative Innovation Center for Diagnosis and Treatment of Infectious Diseases, The First Affiliated Hospital, College of Medicine, Zhejiang University, Hangzhou, China

**Keywords:** HCC, DNMT1, m^5^C modification patterns, TME, prognosis

## Abstract

**Background:** 5-Methylcytosine (m^5^C) plays essential roles in hepatocellular carcinoma (HCC), but the association between m^5^C regulation and immune cell infiltration in HCC has not yet been clarified.

**Methods:** In this study, we analysed 371 patients with HCC from The Cancer Genome Atlas (TCGA) database, and the expression of 13 m^5^C regulators was investigated. Additionally, gene set variation analysis (GSVA), unsupervised clustering analysis, single-sample gene set enrichment analysis (ssGSEA), correlation analysis, and immunohistochemical (IHC) staining were performed.

**Results:** Among the 371 patients, 41 had mutations in m^5^C regulators, the frequency of which was 11.26%. Compared with normal hepatic tissues, the expression of m^5^C regulators with copy number variations (CNVs) expansion was significantly higher than that in HCC tissues. Then, we identified three m^5^C modification patterns that had obvious tumour microenvironment (TME) cell infiltration characteristics. The prognostic analysis of the three major m^5^C modification subtypes showed that Cluster-2 had a clear survival advantage over the others. In addition, we found that DNMT1 was highly expressed in tumour tissues compared with normal tissues in a tissue microarray (TMA) and that it was positively correlated with many TME-infiltrating immune cells. High expression of the m^5^C regulator DNMT1 was related to a poor prognosis in patients with HCC. Furthermore, we developed three distinct Immu-clusters. Importantly, mRNAs related to the transcription of growth factor β (TGF-β)/EMT pathway were significantly up-regulated in Immu-cluster 2, indicating that this cluster is considered to be the immune rejection phenotype. Immu-cluster 3 showed elevated expression of mRNAs related to immune checkpoint genes.

**Conclusion:** Our work revealed the association between m^5^C modification and immune regulators in the TME. These findings also suggest that DNMT1 has great potential as a prognostic biomarker and therapeutic target for HCC.

## Introduction

Hepatocellular carcinoma (HCC) is the sixth most common cancer and the fourth leading cause of cancer-related death worldwide (Llovet et al., 2021). Risk factors for HCC include hepatitis B virus (HBV), hepatitis C virus (HCV), non-alcoholic fatty liver disease, obesity with diabetes, etc. Patients who are infected with HCV can be treated with antiviral therapies, while patients who are infected with HBV remain infected throughout life (European Association for the Study of the Liver, 2017). The survival of patients is driven by tumour stage, with a 5-year survival rate exceeding 70% for those with early-stage HCC compared to a median survival time of 1–1.5 years for those with advanced-stage HCC (Llovet et al., 2016). Most HCC patients are diagnosed at advanced stages, and limited effective therapeutic strategies are available (Hernandez-Gea et al., 2013).

Tumour cells are the driving cause of tumour development and progression. However, without the tumour microenvironment (TME), tumour cells cannot act alone in the progression of cancer. The TME includes the surrounding blood vessels, fibroblasts, immune cells, extracellular matrix, and signalling molecules. These elements contribute to the processes of carcinogenesis and progression, while it is still a major challenge to fully evaluate the complex TME (Hanahan and Coussens, 2012).

Epigenetic deregulation, such as aberrant DNA methylation and reversible chemical RNA modifications play a critical role in cancer (Davalos et al., 2018; García-Vílchez et al., 2019). Previous studies have mainly focused on m^6^A modification in regulating coding and non-coding RNA processing and function (Nombela et al., 2021). Emerging evidence has revealed the important role of 5-methylcytosine (m^5^C) in posttranscriptional regulation (Xue et al., 2020a). In addition, m^5^C modification was found to be abundant in mammalian cells, characterised by the addition of a methyl group at the carbon-5 position of the cytosine base (Bestor, 1988). m^5^C is mainly distributed in GC-rich areas. Over 10,000 potential sites of m^5^C modification have been detected in the whole human transcriptome (Bourgeois et al., 2015). The regulation of m^5^C is a dynamic process controlled by three major regulators, termed “writers” (add a special modification), “readers” (identify and bind modified nucleotides), and “erasers” (remove a special modification) (Yang et al., 2018).

Recently, targeting the TME has been an encouraging method for cancer treatment (Bejarano et al., 2021). Some studies showed a correlation between m^6^A and TME-infiltrating immune cells (Yi et al., 2020; Zhang et al., 2020a; Chong et al., 2021; Shen et al., 2021). However, due to technological limitations, the research above was restricted to one or two type of modification regulators or cell types, while anti-tumour effects involve multiple tumour suppressors interacting in a vitally cooperative way. Hence, a deep understanding of TME cell infiltration mediated by several regulators of gene modifications will help to enhance the perception of TME immune regulation, especially m^5^C modification.

In this study, we analysed 371 patients with HCC from The Cancer Genome Atlas (TCGA) database, and the samples were integrated to evaluate m^5^C modification patterns. Correlation analysis was performed between the m^5^C modification pattern and TME cell infiltration characteristics. Three different m^5^C modification patterns were discovered based on the expression of 13 m^5^C regulators. Besides, we found that distinct m^5^C modification patterns were closely associated with different enrichment pathways and immune cell infiltration characteristics, indicating that m^5^C modification might play an essential role in forming an individual TME.

## Materials and Methods

### HCC Data Source and Preprocessing

Gene expression and clinical annotation data were downloaded from the TCGA database. Patients without complete survival data were excluded. The TCGA-Liver Hepatocellular Carcinoma (TCGA-LIHC) dataset was used for further analysis. Finally, a total of 371 patient in the TCGA-LIHC cohort were selected for this study.

For the TCGA dataset, the R package TCGA biolinks (Colaprico et al., 2016), which was developed to analyse Genomic Data Commons (GDC) data, was utilised to download the fragments per kilobase per million mapped reads (FPKM) values of gene expression from the GDC.^1^ FPKM values were further converted to transcripts per kilobase million (TPM) values. Batch effects generated by factors unrelated to any biological variations were corrected for using the parametric and non-parametric empirical Bayes framework algorithm from the sva package. Data related to somatic mutations were downloaded from the TCGA database. R (3.6.1) together with Bioconductor packages were employed in the study.

### Unsupervised Clustering Analysis of m^5^C Regulators

A total of 13 m^5^C regulators were extracted from 371 patients in the TCGA-LIHC cohort: 11 writers (NOP2, NSUN2, NSUN3, NSUN4, NSUN5, NSUN6, NSUN7, DNMT1, TRDMT1, DNMT3A, and DNMT3B), 1 eraser (TET2), and 1 reader (ALYREF). Unsupervised clustering analysis was employed to distinguish different m^5^C modifications, after which the classification of patients was conducted for subsequent analysis.

A consensus clustering algorithm (Hartigan and Wong, 1979) was employed to assure the number of clusters and their stability. The ConsensusClusterPlus package was applied to execute the workflow mentioned above, and the stability of the classification was accomplished by conducting 1000 repetitions (Wilkerson and Hayes, 2010).

### Gene Set Variation Analysis and Functional Annotation

To explore the disparity of biological processes in m^5^C modification patterns, the gene set variation analysis (“GSVA”) R package was used to perform GSVA. This package is based on a non-parametric and unsupervised algorithm and is widely used to estimate the variation in gene set enrichment in expression datasets (Hänzelmann et al., 2013). GSVA was implemented with “c2.cp.kegg.v6.2.symbols” gene sets obtained from the Molecular Signatures Database (MsigDB). An adjusted *P*-value of less than 0.05 was regarded as statistically significant. We applied the “ClusterProfiler” R package to functionally annotate m^5^C-related genes under the false discovery rate (FDR) threshold of <0.05.

### Single-Sample Gene Set Enrichment Analysis

The single-sample gene set enrichment analysis (ssGSEA) algorithm was used to determine the relative richness in cell infiltration in the TME. We obtained the gene set associated with each infiltrating immune cell type in the TME from Charoentong, who stores information on various human immune cells, including CD8 T cells, dendritic cells (DCs), natural killer (NK) T cells, macrophages, regulatory T cells, etc. (Barbie et al., 2009; Charoentong et al., 2017). ssGSEA was employed to determine the enrichment scores and define the relative abundance of each TME-infiltrating cell type in the corresponding sample.

### Identification of Differentially Expressed Genes Among the m^5^C Phenotypes

With the aim of distinguishing m^5^C-related genes, all the patients were divided into three m^5^C modification patterns according to the expression of m^5^C regulators. The empirical Bayesian algorithm under the limma package in R was used to assure differentially expressed genes (DEGs) in heterogeneous modification patterns.

### Correlation Between the m^5^C Gene Signature and Biological Pathways

A set of genes was constructed by Mariathasan et al. (2018), Rosenberg et al. (2016) and Şenbabaoğlu et al. (2016), in which genes associated with certain biological processes are stored. Correlation analysis was employed to explore the association between the gene signature of m^5^C and biological pathways.

### Cell Culture

Human liver cell line Huh7 and paired normal human liver cell L02 were purchased from Chinese Academy of Sciences (Shanghai, China) and cultured in DMEM (Gibco, Carlsbad, CA, United States) supplemented with 10% fetal bovine serum (FBS; Gibco; Thermo Fisher Scientific) and 1% penicillin n (MP Biomedicals, Santa Ana, CA, United States). The cells were cultured at 37°C in atmosphere of 5% CO_2_.

### Quantitative Reverse-Transcription PCR

Total RNA from Huh7 cell line was extracted with Rneasy Mini Kit (Qiagen, Valencia, CA, United States) and then reverse-transcribed into cDNA preformed using the PrimeScript^TM^ RT reagent Kit. GAPDH was used as the internal control. The expression levels of 11 writers (NOP2, NSUN2, NSUN3, NSUN4, NSUN5, NSUN6, NSUN7, DNMT1, TRDMT1, DNMT3A, and DNMT3B), 1 eraser (TET2), and 1 reader (ALYREF) were quantified using 2^−ΔΔCt^ method by ABI7500fast PCR instrument. The primers are listed in Supplementary Table 1.

### Immunohistochemical Staining

Human HCC tissue arrays and normal tissues (catalogue number: HlivH180Su15) were purchased from Shanghai Outdo Biotech Co., Ltd. (Shanghai, China). The method of immunohistochemical (IHC) staining has been reported previously. Briefly, antigen retrieval was performed by heating the tissue sections at 100°C for 30 min in target retrieval solution. Then, the tissue microarray (TMA) was incubated with a DNMT1 primary antibody [(EPR18453) (ab188453) Abcam, Cambridge, MA, United States], followed by incubation with an anti-rabbit secondary antibody. Two independent pathologists blindly assessed the IHC results according to the staining area and intensity (Zhang et al., 2020b).

### Statistical Analysis

Spearman and distance correlation analyses were performed to obtain the correlation coefficients of the TME-infiltrating immune cells and the corresponding expression of m^5^C regulators. Student’s *T*-test was used for comparisons two groups. One-way analysis of variance (ANOVA) and Kruskal–Wallis tests were performed to compare differences between three or more groups (Hazra and Gogtay, 2016). The Kaplan–Meier method was utilised to generate survival curves for the prognostic analysis, and the log-rank test was applied to identify significant differences. Univariate Cox regression was adopted to determine the hazard ratios of m^5^C regulators and genes related to specific m^5^C phenotypes. Multivariable Cox regression was utilised to identify independent prognostic risk factors. Patients with complete relevant data were subjected to further analysis with a multivariate model. The multivariate results were visualised with the forestplot package in R. Copy number variations (CNVs) in 13 m^5^C regulators were plotted with the Rcircos package (Mayakonda et al., 2018). All *P*-values were two-sided, with *P* < 0.05 considered statistically significant. The analysis was accomplished in R 3.6.1 software.

## Results

### Landscape of Genetic Variations in m^5^C Regulators in HCC

**FIGURE 1:**
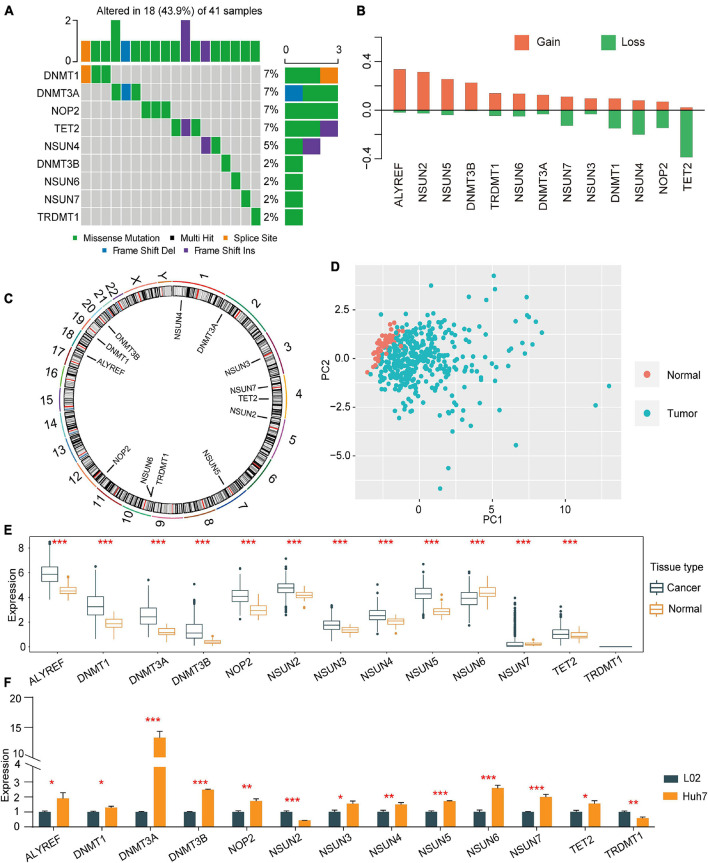
Copy number variations and somatic mutations in 13 m^5^C regulators in HCC. **(A)** Mutation frequencies of the top 9 m^5^C regulators. **(B)** CNV alterations among the 13 regulators. **(C)** Locations of mutations in the m^5^C regulators at the chromosome level. **(D)** Principal component analysis was used to distinguish tumour tissues and normal tissues based on the expression of m^5^C regulators. **(E)** The expression profiles of m^5^C regulator genes in tumour tissues and adjacent normal tissues. **(F)** qRT-PCR was used to determine the relative expression of NOP2, NSUN2, NSUN3, NSUN4, NSUN5, NSUN6, NSUN7, DNMT1, TRDMT1, DNMT3A, DNMT3B, TET2, and ALYREF in HCC cell line Huh7 and normal control cell line L02. *p < 0.05, **p < 0.01, ***p < 0.001.

A total of 13 regulators of m^5^C were identified, including 11 writers, 1 eraser, and 1 reader. First, the incidence of CNVs and somatic mutations in regulators in HCC were summarised. In 364 samples, 41 showed mutations in m^5^C regulators, the occurrence of which was 11.26%. DNMT1 was found to be exposed to a higher frequency of mutations, followed by DNMT3A, while ALYREF, NSUN2, NSUN3, and NSUN5 were not (Figure 1A). CNVs were also detected in 13 other regulators upon exploration of their modification frequencies. Most of the modifications involved a copy number expansion, but TET2, NOP2, and NSUN4 had a broad occurrence of deletions (Figure 1B). The chromosome sites of the m^5^C regulators are shown in Figure 1C. Based on the expression of 13 m^5^C regulators in HCC patients, HCC samples could be thoroughly differentiated from normal samples (Figure 1D). To determine whether the expression of m^5^C regulators was influenced by the genetic mutations mentioned above, the mRNA expression of regulators was explored. We found that a change in m^5^C was an important factor leading to perturbations in the expression of m^5^C regulators. Compared with normal hepatic tissues, the expression of m^5^C regulators with a CNV expansion was significantly higher than that in HCC tissues (e.g., ALYREF and NSUN2) (Figures 1B,E). In addition, the expression of in HCC cell line Huh7 and normal control cell line L02 were detected by quantitative reverse-transcription PCR (qRT-PCR). Assistant with the expression in TCGA, the expression of ALYREF, DNMT1, DNMT3A, DNMT3B, NOP2, NSUN3, NSUN4, NSUN5, NSUN6, NSUN7, and TET2 were higher in HCC cell line Huh7 than in normal cell line L02. While the expression level of TRDMT1 and NSUN2 was lower in Huh7 than in L02 (Figure 1F). The analyses above showed that the genetic and expression alteration landscape of m^5^C regulators in normal tissues and HCC tissues is highly heterogeneous, suggesting that the expression imbalance of m^5^C regulators plays an important role in HCC occurrence and progression.

### m^5^C Methylation Alteration Patterns Mediated by 13 Regulators

**FIGURE 2:**
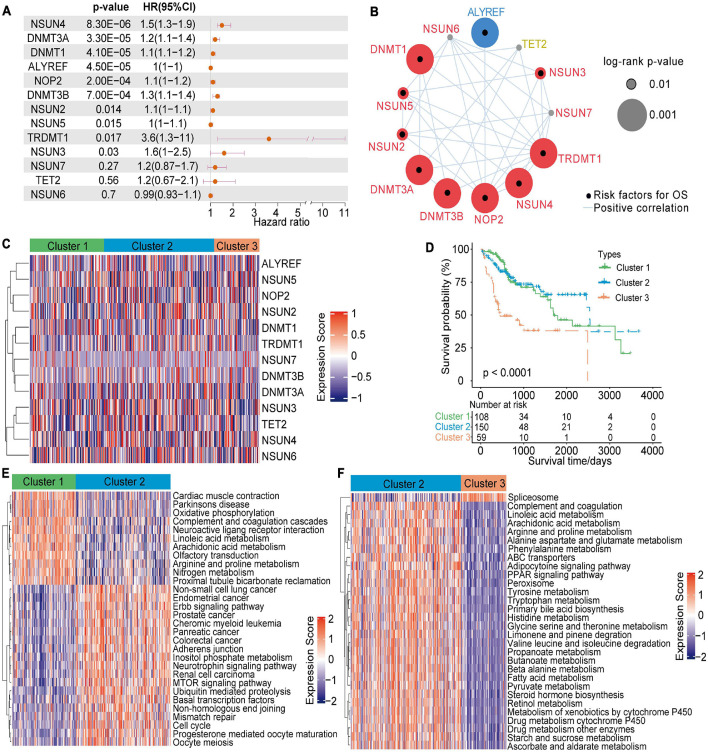
5-Methylcytosine methylation alteration patterns and related biological characteristics. **(A)** Univariate Cox regression analysis of the 13 m^5^C regulators in patients with HCC. **(B)** The network of m^5^C regulators and their prognostic significance for HCC patients. **(C)** Unsupervised clustering analysis of 13 m^5^C regulators in HCC. **(D)** Survival analysis of HCC patients in the TCGA-LIHC cohort according to the three m^5^C clusters. **(E,F)** A heatmap of GSVA results shows the representative hallmark pathways associated with distinct m^5^C modification patterns.

Univariate Cox regression analysis showed that 13 m^5^C modulators have prognostic significance in HCC patients (Figure 2A). The m^5^C regulator network revealed m^5^C modulator interactions, modulator connections and their prognostic significance for patients (Figure 2B). The R package Consensus Cluster Plus was applied to classify patients with qualitatively different m^5^C alteration patterns according to the expression of 13 m^5^C regulators, and unsupervised clustering analysis was performed to identify a total of 3 different modification patterns (120 cases in modification pattern 1, 178 cases in modification pattern 2, and 73 cases in modification pattern 3; referred to as m^5^C Clusters 1–3, respectively) (Figure 2C and Supplementary Table 2). The prognostic analysis of the three major m^5^C modification subtypes showed that Cluster-2 had a clear survival advantage over the others (Figure 2D). The above results indicate that the regulators of m^5^C may play an important role in m^5^C alteration patterns and TME cell infiltration characteristics between individual tumours.

### TME Cell Infiltration Characteristics in Different m^5^C Modification Patterns

**FIGURE 3:**
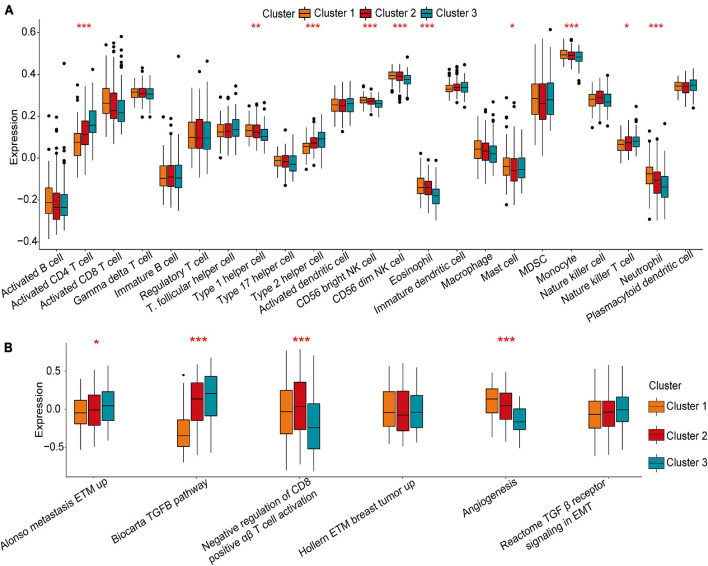
Tumour microenvironment characteristics in different m^5^C modification patterns. **(A)** Comparison of the abundance of immune infiltrating cells in three clusters. **(B)** Differences in cellular biological pathways among the three clusters. *p < 0.05, **p < 0.01, ***p < 0.001.

To investigate the biological actions associated with m^5^C modification patterns, GSVA was conducted. As shown in Figure 2E and Supplementary Table 2, m^5^C Cluster-2 was remarkably enriched in carcinogenesis pathways, such as the ERBB signalling pathway, cell cycle signalling pathway, and adherens junction pathway. Cluster-1 was associated with many metabolism pathways, such as, oxidative phosphorylation, linoleic acid metabolism, arachidonic acid metabolism, arginine and proline metabolism, and nitrogen metabolism (Figure 2E). Cluster-3 was highly associated with spliceosome (Figure 2F). Further analysis of TME cell infiltration showed that Cluster-1

was significantly enriched in the infiltration of innate immune cells, including eosinophils, NK cells, macrophages, CD8 T cells, and mast cells (Figure 3A). Prior research has shown that tumours with an immune rejection phenotype exhibit large amounts of immune cells, and these immune cells are in the matrix around the tumour cell nest instead of inside the tissue (Chen and Mellman, 2017). GSVA showed that the modification of Cluster-1 was significantly related to matrix activation. Therefore, it was speculated that the Cluster-1 matrix serves as an activation inhibitor of the anti-tumour effect of immune cells. Further analysis showed that matrix activity was greatly upgraded in Cluster 1, activating the angiogenesis pathway. These results supported our hypothesis (Figure 3B).

### The m^5^C Regulator DNMT1 Has a Strong Relationship With Infiltrating Immune Cells

**FIGURE 4:**
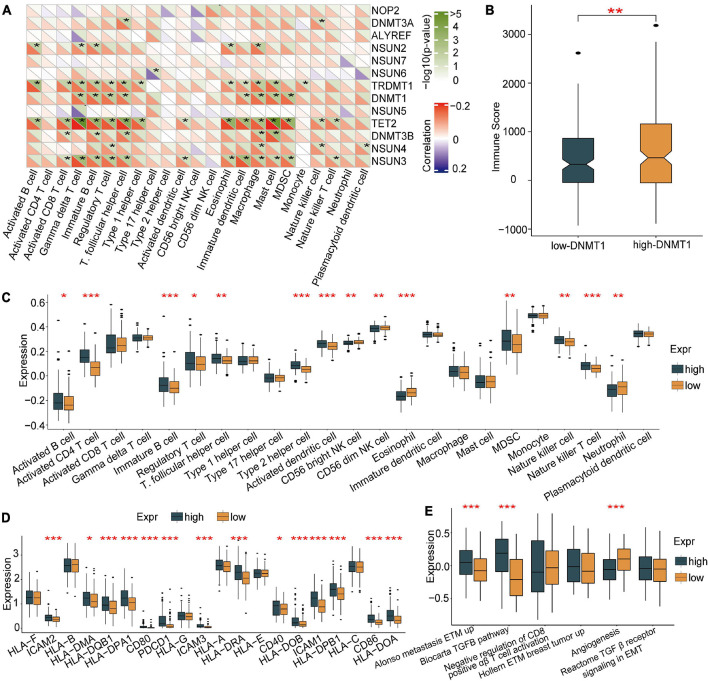
Association of TME-infiltrating cells with the m^5^C regulator of DNMT1. **(A)** Correlation between m^5^C regulators and different immune cells using Spearman analysis. **(B)** Immune scores of the low DNMT1 group and the high DNMT1 group. **(C)** Comparison of the abundance of immune-infiltrating cells in the low DNMT1 group and high DNMT1 group. **(D)** Correlation between m^5^C regulators and the activation of dendritic cells. **(E)** High DNMT1 expression shows significant enhancement of the immune-activated pathway. *p < 0.05, **p < 0.01, ***p < 0.001.

To further explore the role of each m^5^C regulator in the TME, Spearman correlation analysis was applied to examine the correlation between each TME-infiltrating cell type and m^5^C regulators (Figure 4A). An emphasis was placed on the regulator DNMT1, an m^5^C methyltransferase, and we revealed its positive relationship with the infiltration of many TME immune cells. An estimation method was applied to determine the expression of DNMT1 and the infiltration of immune cells. The results showed that higher DNMT1 expression was related to a higher immune score, which means that a TME with high DNMT1 expression has significantly high immune cell infiltration (Figure 4B). Based on these results, the specific differences in 23 TME-infiltrating immune cells were explored between patients with high and low DNMT1 expression. We found that tumours exhibiting high DNMT1 expression had markedly more infiltration of 13 TME immune cells than those exhibiting low expression (Figure 4C). Recently, attention was drawn to the regulatory mechanisms of m^5^C modification on the activation of DCs, which are the bridge connecting innate immunity with adaptive immunity, the activation of which depends on upregulating the expression of MHC molecules, adhesion molecules, and costimulatory molecules (Figure 4D). As expected, subsequent enrichment analysis showed that tumours with high DNMT1 expression showed remarkable enrichment in immune activation pathways (Figure 4E). Therefore, it was speculated that m^5^C methylation modification mediated by DNMT1 may contribute to activated DCs in the TME, thus promoting the anti-tumour immune response in HCC.

### High Expression of the m^5^C Regulator DNMT1 in Tumour Tissues Is Related to a Poor Prognosis in Patients With HCC

**FIGURE 5:**
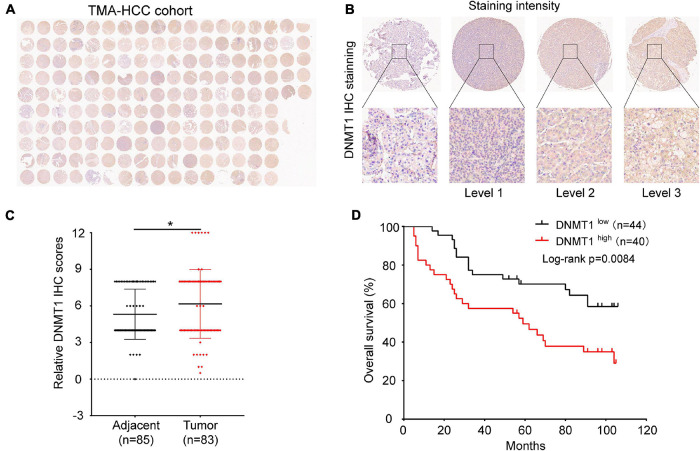
Expression of DNMT1 in human HCC tumour tissues and control tissues. **(A)** Panoramic scanning of DNMT1 by IHC staining. **(B)** Representative IHC staining of DNMT1 in samples. **(C)** The expression of DNMT1 is higher in HCC tissues than in normal tissues. **(D)** Kaplan–Meier analysis showed that patients with higher levels of DNMT1 had shorter OS times than those with low levels of DNMT1. *p < 0.05.

Immunohistochemical staining was used to determine the expression pattern of DNMT1 on a TMA consisting of 90 pairs of HCC tissues and adjacent tissues. Representative micrographs illustrate the various degrees of DNMT1 expression (Figures 5A,B). The expression of DNMT1 was higher in tumour tissues than in control tissues (Figure 5C), which was consistent with the findings in the TCGA-LIHC cohort (Figure 1E). The correlation of DNMT1 expression with the clinicopathological characteristics of patients with HCC is shown in Supplementary Table 3. In addition, Kaplan–Meier curve analysis showed that patients with high DNMT1 expression had shorter overall survival (OS) than those with low DNMT1 expression (Figure 5D). Univariable and multivariable Cox regression analyses were used to determine whether the expression of DNMT1 was an independent risk factor. The univariable analysis revealed that DNMT1 expression was associated with tumour size and TB, AFP, and PD-L1 levels (*P* < 0.05, Supplementary Table 4). Further analysis demonstrated that DNMT1 might serve as a prognostic predictor for HCC.

### Generation of the m^5^C Gene Signature and Functional Annotation

**FIGURE 6:**
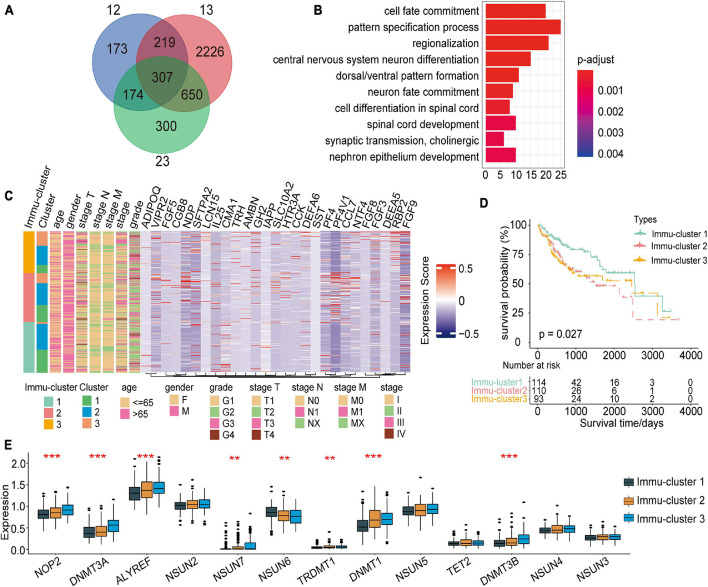
Identification of distinct Immu-clusters based on immune-related DEGs in m^5^C modification patterns. **(A)** A total of 307 m^5^C-related DEGs between three m^5^C clusters were identified, as shown in the Venn diagram. **(B)** Enrichment of biological processes significantly related to DEGs. **(C)** The selected genes were used to classify patients into different genomic subtypes by unsupervised clustering analysis. **(D)** Kaplan–Meier curves indicated that the genomic subtypes were correlated with the prognosis of patients with HCC. **(E)** Significant differences in the expression of m^5^C regulators. **p < 0.01, ***p < 0.001.

For subsequent exploration of the biological behaviour of each m^5^C modification pattern, we ascertained 307 m^5^C phenotype-related DEGs with the limma package (Figure 6A). Cluster profiler was employed to implement enrichment analysis on the DEGs. Supplementary Table 5 summarises the significantly enriched pathways. As expected, we detected enrichment in biological processes that are notably related to m^5^C modification and immunity, which verified the important role that m^5^C modification plays in immune regulation in the TME (Figure 6B).

To further explain the association, we performed unsupervised clustering analysis to classify 307 m^5^C phenotype-related genes and extracting 27 immune-related genes: VIPR2, CCL7, RBP2, SLC10A2, FGF5, DEFA5, HTR3A, TRH, LCN15, AMBN, ADIPOQ, FGF3, CCK, NTF4, NDP, FGF9, PF4, CMA1, SFTPA2, CGB8, DEFA6, PF4V1, IL25, GH2, FGF8, SST, and

IAPP. Furthermore, we performed unsupervised clustering analysis based on these genes to categorise patients into different subtypes (Supplementary Figures 1A–D). In line with the clustering analysis of m^5^C modification patterns, unsupervised clustering analysis revealed three different m^5^C-modified phenotypes termed Immu-clusters 1–3, respectively. Thus, there are three different distinct immune-related m^5^C methylation patterns. We observed that tumours in Immu-clusters 2 and 3 were associated with poor differentiation and enriched in diffuse histological subtypes. The opposite pattern was observed in Immu-cluster 1. Patients whose survival status was known were mainly concentrated in Immu-cluster 1, while patients in clinical stage IV or with a high TNM grade were mainly concentrated in Immu-cluster 2 (Figure 6C). The analysis also showed that three different gene clusters had different feature genes (Figure 6C). In total, 114 of the 317 HCC patients clustered in Immu-cluster 1, which was associated with a better prognosis. The prognosis of patients in Immu-cluster 2 (110 patients) and Immu-cluster 3 (93 patients) was poor (Figure 6D). In the three immune clusters, a significant distinction in the expression of m^5^C regulatory factors emerged. This result was consistent with the m^5^C methylation modification patterns (Figure 6E).

### Clinical and Transcriptional Features of the m^5^C-Related Phenotypes

**FIGURE 7:**
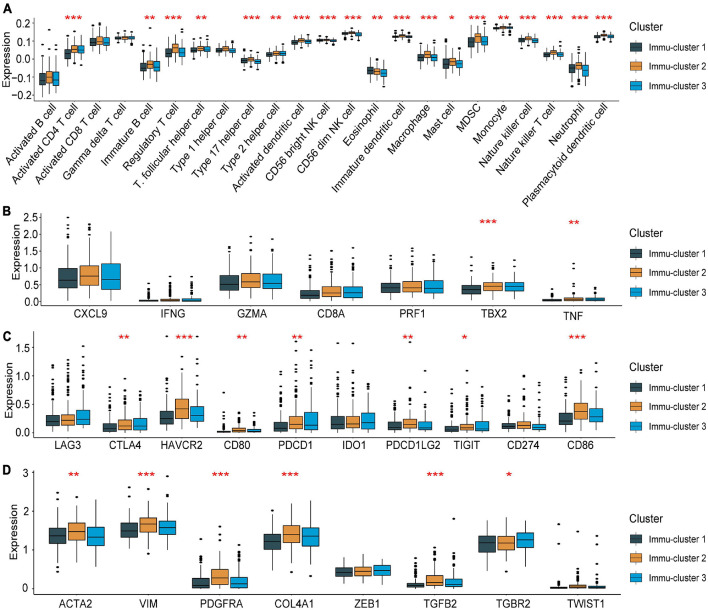
Association between the expression of m^5^C regulators and immunoregulation in the TME. **(A)** Differences in immune cell infiltration in the three Immu-clusters. **(B)** Comparison of immune-related cytokine expression in the three Immu-clusters. **(C)** Comparison of the transcription of immune checkpoint genes in the three Immu-clusters. **(D)** Immu-clusters involved in the transcription of the TGF-β/EMT pathway. *p < 0.05, **p < 0.01, ***p < 0.001.

To further explain the role that m^5^C-related phenotypes play in TME immune regulation, the levels of immune cells and expression of chemokines and cytokines in the three Immu-clusters were examined. The chosen cytokines and chemokines were taken from previously existing studies (Turley et al., 2015). Our analysis showed that activated CT4 T cells, immature B cells, regulatory T cells, NK cells, macrophages, mast cells, myeloid-derived suppressor cells (MDSCs), monocytes, neutrophils, and plasmacytoid DCs were significantly different among the Immu-clusters. Besides, the immunosuppressive cells (including MDSCs and regulatory T cells) were significantly upregulated in Immu-cluster 2 (Figure 7A). Tumour necrosis factor, interferon, CD8A, CXCL9, CXCL10, GZMA, GZMB, PRF1, and TBX2 were associated with immune activation transcription (Barbie et al., 2009; Zeng et al., 2019). The expression of TNF and TBX2 were different in this three Immu-clusters (Figure 7B). PD-L1, CD80, CD86, CTLA-4, HAVCR2, etc., were thought to be related to the transcription of immune checkpoints. We compared the transcription of these immune checkpoint genes in the three Immu-clusters and found that the expression of most of the immune checkpoint genes were remarkedly different (Figure 7C). ACTA2, CLDN3, VIM, COL4A1, SMAD9, TWIST1, TGFBR2, TGRB1, and ZEB1 are related to the transcription of growth factor β (TGF-β)/EMT pathway transformation and exhibited significant differences between the three Immu-clusters (Figure 7D). We found that mRNAs related to the TGF-β/EMT pathway were significantly upregulated in Immu-cluster 2, indicating that this cluster is the matrix-activated group and associated with immunosuppression. Immu-cluster 3 showed elevated expression of mRNAs related to immune checkpoint genes, suggesting that the patients in this group may respond better to immune checkpoint drugs, which requires further study.

## Discussion

According to previous reports, tumours, including HCC, are mainly driven by genetic mutations. In recent years, epigenetic modifications have been found to play a critical role in the carcinogenesis and molecular pathogenesis of HCC (Xue et al., 2020a; Nombela et al., 2021). m^5^C is the most preventative and best understood DNA modification in eukaryotes (Piperi and Papavassiliou, 2011). In recent years, emerging evidence has revealed the important role of RNA m^5^C in posttranscriptional regulation. Several studies have revealed that m^5^C regulators and m^5^C methylation play essential roles in different cancer types, including HCC. He et al. (2020b) found that ALYREF and NSUN4 could be promising targets for HCC therapies. In addition, studies showed the map of m^5^C methylation based on HCC tissues and paired non-tumour tissues at the mRNA, lncRNA, and circRNA levels (He et al., 2020a,c; Zhang et al., 2020c). Recent studies showed that NSUN2 could promote tumour progression in HCC (Sun et al., 2020) and gastric cancer (Mei et al., 2020). Similar to our findings, Cui et al. (2021) and Xue et al. (2020b) found that DNMT1 played important roles in head and neck squamous cell carcinoma.

Recently, increasing evidence has shown interactions between the tumour immune-microenvironment (TIME) and m^6^A modifications. Yi et al. (2020) reported that copy number alterations in m^6^A methylation regulators affected immune cell infiltration in head and neck squamous cell carcinoma. Lin et al. (2020) also attempted to explore the relationship between m^6^A regulators and tumour-infiltrating immune cells by ssGSEA in glioma. Shen et al. (2021) found that m^6^A modification patterns were correlated with immune regulation in HCC and might provide novel immune therapeutic targets. However, as an important epigenetic modification, the role of m^5^C methylation in the immune regulation of HCC is still unclear. Here, we described the TME cell infiltration characteristics in different m^5^C modification patterns. Furthermore, we identified three distinct immune-related m^5^C methylation subtypes and investigated the levels of immune cells and expression of chemokines and cytokines in the three Immu-clusters. All the results indicate that the generation of immune-related m^5^C methylation subtypes contribute to understanding the molecular mechanisms of HCC and provide novel clues for predicting the prognosis of patients with HCC.

It has been demonstrated that DNMT1 is an essential methyltransferase for the maintenance of DNA methylation. Previous evidence has shown that DNMT1 is overexpressed in breast cancer (Wang et al., 2018), thyroid cancer cells (Zhang et al., 2018), and pancreatic cancer (Peng et al., 2005). Furthermore, high DNMT1 expression is significantly associated with a poor prognosis (Li et al., 2010; Hong et al., 2018). Consistent with our results, we found that DNMT1 expression was increased in tumour tissues compared with normal tissues in the TMA and TCGA cohort. In our study, Kaplan–Meier curve analysis and univariable and multivariable Cox regression analysis further demonstrated that the expression of DNMT1 is an independent risk factor for HCC. Therefore, DNMT1 might serve as a promising prognostic predictor and therapeutic target for HCC.

## Conclusion

Taken together, our results showed the association between m^5^C modification and TME. Moreover, we found a key m^5^C modification regulator, DNMT1, which has great potential as a prognostic biomarker and therapeutic target for HCC.

## Data Availability Statement

The datasets presented in this study can be found in online repositories. The names of the repository/repositories and accession number(s) can be found in the article/Supplementary Material.

## Ethics Statement

The studies involving human participants were reviewed and approved by The First Affiliated Hospital, College of Medicine, Zhejiang University. The patients/participants provided their written informed consent to participate in this study.

## Author Contributions

XG and HZo designed the whole study. QZ and JW conducted the statistical analysis. XG and QC draft the manuscript. QC made the relevant edits to the manuscript. XG and HZu revised the manuscript. All authors read and approved the final manuscript.

## Conflict of Interest

The authors declare that the research was conducted in the absence of any commercial or financial relationships that could be construed as a potential conflict of interest.

## Publisher’s Note

All claims expressed in this article are solely those of the authors and do not necessarily represent those of their affiliated organizations, or those of the publisher, the editors and the reviewers. Any product that may be evaluated in this article, or claim that may be made by its manufacturer, is not guaranteed or endorsed by the publisher.

## Footnotes

1
https://portal.gdc.cancer.gov

## Supplementary Material

Supplementary Figure 1 Consensus map of unsupervised clustering results used for subtype analysis.

Supplementary Table 1 The primers for the qRT-PCR assay.

Supplementary Table 2 Estimating the relative abundance of tumour cells in the TME in the TCGA-LIHC cohort by single-sample gene set enrichment analysis (ssGSEA).

Supplementary Table 3 Activation states of biological pathways in distinct m^5^C modification patterns by GSVA.

Supplementary Table 4 Correlation between DNMT1 expression and clinicopathological characteristics.

Supplementary Table 5 Univariate and multivariate analyses of the factors correlated with the OS of HCC patients.

Supplementary Table 6 Functional annotation of m^5^C phenotype-related genes.

## Abbreviations

m^5^C: 5-methylcytosine
HCC: hepatocellular carcinoma
TCGA: The Cancer Genome Atlas
GSVA: gene set variation analysis
ssGSEA: single-sample gene set enrichment analysis
IHC: immunohistochemical
TME: tumour microenvironment
TMA: tissue microarray
HBV: hepatitis B virus
HCV: hepatitis C virus
m^6^A: *N*^6^-methyladenosine
GDC: Genomic Data Commons
FPKM: fragments per kilobase per million mapped reads
TPM: transcripts per kilobase million
MsigDB: Molecular Signatures Database
FDR: false discovery rate
DCs: dendritic cells
NK: natural killer
DEGs: differentially expressed genes
ANOVA: one-way analysis of variance
CNVs: copy number variations
OS: overall survival
MDSCs: myeloid-derived suppressor cells
TIME: tumour immune-microenvironment.
