# Supplementary figures and images for: Uncovering the Association Between m5C Regulator-Mediated Methylation Modification Patterns and Tumour Microenvironment Infiltration Characteristics in Hepatocellular Carcinoma
Source: Front Cell Dev Biol. 2021 Sep 13;9:727935. doi: 10.3389/fcell.2021.727935 (PMC8475949; doi:10.3389/fcell.2021.727935)

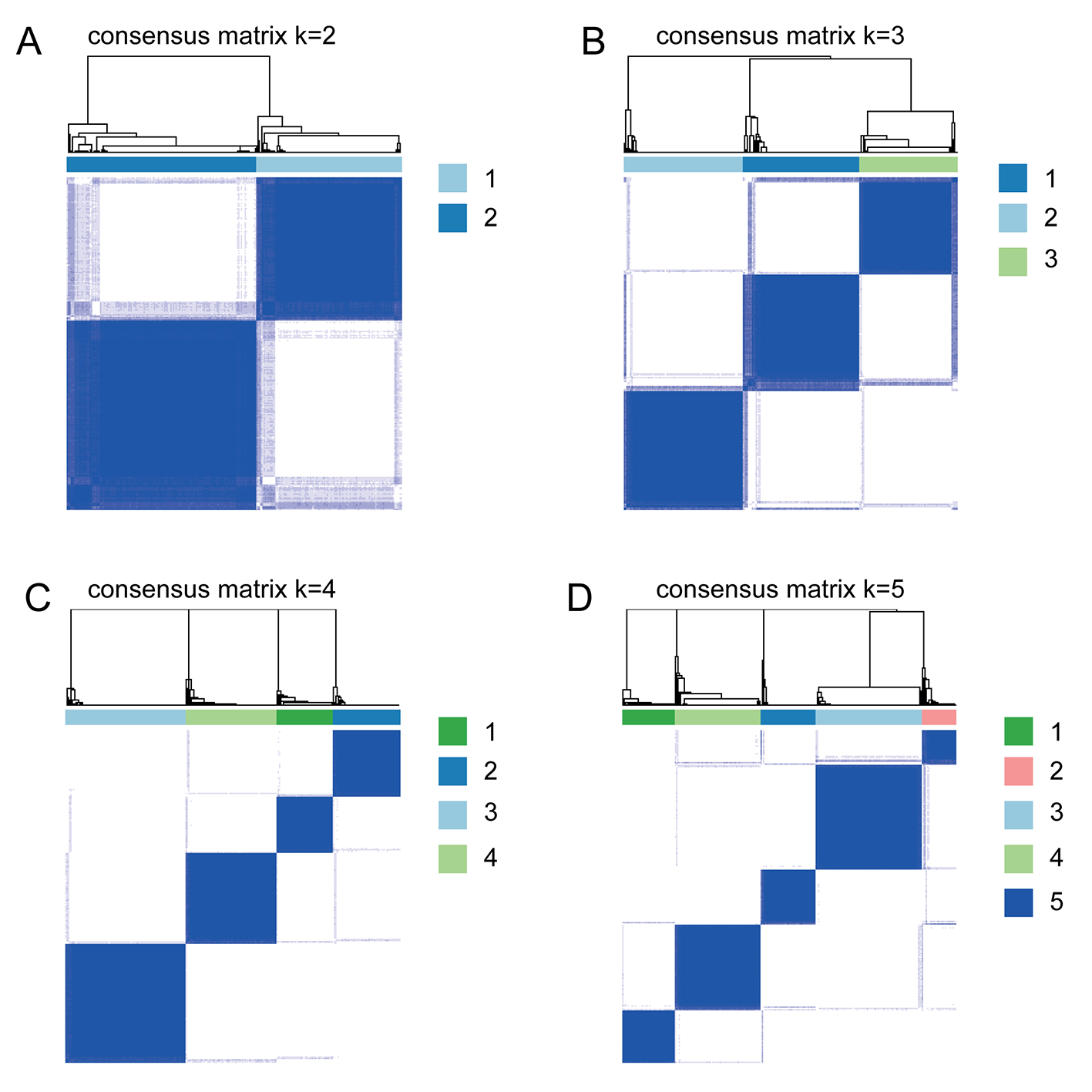

Supplement: Supplementary Figure 1 — Consensus map of unsupervised clustering results used for subtype analysis. [file Image_1.TIF]
