# Supplementary material for: Uncovering the Association Between m5C Regulator-Mediated Methylation Modification Patterns and Tumour Microenvironment Infiltration Characteristics in Hepatocellular Carcinoma
Source: Front Cell Dev Biol. 2021 Sep 13;9:727935. doi: 10.3389/fcell.2021.727935 (PMC8475949; doi:10.3389/fcell.2021.727935)
Supplement: Supplementary Table 1 — The primers for the qRT-PCR assay. [file Table_1.docx]

**Supplementary Table 1.** The primers for the qRT-PCR assay**.**

| NOP2(human)-F | GTGTTGTGGGCAACTTGCAT |
| --- | --- |
| NOP2(human)-R | TCCTTGGAGATGACCCCAGT |
| NSUN2(human)-F | GTGAAGGTGCTTTGGAGCTTG |
| NSUN2(human)-R | GAGGGAACATGGTAGGTCGG |
| NSUN3(human)-F | GCTAAGGCAGACGTTGGAATC |
| NSUN3(human)-R | TTTGAACACGGAGCATCCAC |
| NSUN4(human)-F | CTCTCGGTCCTGTCTCCA |
| NSUN4(human)-R | AACCTGCTGATGCTCCAC |
| NSUN5(human)-F | AAGCATTTTCTCCTGGACCCC |
| NSUN5(human)-R | GGCAAAGATCTTCCCTTGGTT |
| NSUN6(human)-F | GTGGATTACCTGAACTGAAAGGC |
| NSUN6(human)-R | GTTGTTTTCCCTCCAGGTGC |
| NSUN7(human)-F | TTGCTGAGTTCCTGAATCG |
| NSUN7(human)-R | TTGGGTTTTCTGGTCTGTG |
| DNMT1(human)-F | AAGTGGGGGACTGTGTCTCT |
| DNMT1(human)-R | CGTGAAACATCTGCCCGTTG |
| TRDMT1(human)-F | CTGTTAGACATTGTTCAGCCCA |
| TRDMT1(human)-R | TATTCTCAACCTGCACATCCTCT |
| DNMT3A(human)-F | AAAGCAGGGCAAAGACCA |
| DNMT3A(human)-R | GCGGCTCATGTTGGAGA |
| DNMT3B(human)-F | GGGATGAAGATCAGAGCCGA |
| DNMT3B(human)-R | GTGGAAGGACACGGGGTTTT |
| TET2(human)-F | GGCAGCACAAACACAACCAT |
| TET2(human)-R | GGGGGTGAGGAAAAGTCTGG |
| ALYREF (human)-F | TCTGGTCGCAGCTTAGGAAC |
| ALYREF (human)-R | TGCCACCTCTGTTTACGCTC |
