# Supplementary material for: Uncovering the Association Between m5C Regulator-Mediated Methylation Modification Patterns and Tumour Microenvironment Infiltration Characteristics in Hepatocellular Carcinoma
Source: Front Cell Dev Biol. 2021 Sep 13;9:727935. doi: 10.3389/fcell.2021.727935 (PMC8475949; doi:10.3389/fcell.2021.727935)
Supplement: Supplementary Table 4 — Correlation between DNMT1 expression and clinicopathological characteristics. [file Table_4.docx]

**Supplementary Table 4∣**Correlation between DNMT1 expression and clinicopathological characteristics.

|  | **variables** | **DNMT1 expression** | | **total** | **χ2** | **p value** |
| --- | --- | --- | --- | --- | --- | --- |
|  |  | low | High |  |  |  |
| Age (year) |  |  |  |  | 0.717 | 0.397 |
|  | ≤50 | 18 | 21 | 39 |  |  |
|  | ＞50 | 26 | 21 | 47 |  |  |
| Sex |  |  |  |  | 0.078 | 0.781 |
|  | Female | 5 | 4 | 9 |  |  |
|  | male | 39 | 38 | 77 |  |  |
| Grade |  |  |  |  | 1.175 | 0.278 |
|  | Ι-II | 24 | 18 | 42 |  |  |
|  | Ⅲ | 20 | 24 | 44 |  |  |
| Size |  |  |  |  | 5.811 | 0.016 |
|  | ≤5cm | 25 | 34 | 59 |  |  |
|  | ＞5cm | 19 | 8 | 27 |  |  |
| T stage |  |  |  |  | 0.010 | 0.921 |
|  | T1 | 31 | 30 | 61 |  |  |
|  | T2/T3 | 13 | 12 | 25 |  |  |
| TNM stage |  |  |  |  | 0.010 | 0.921 |
|  | I | 31 | 30 | 61 |  |  |
|  | II/III | 13 | 12 | 25 |  |  |
| Cirrhosis |  |  |  |  | 0.005 | 0.941 |
|  | negative | 4 | 5 | 9 |  |  |
|  | positive | 40 | 37 | 77 |  |  |
| HBsAg |  |  |  |  | 0.442 | 0.506 |
|  | negative | 11 | 8 | 19 |  |  |
|  | positive | 33 | 34 | 67 |  |  |
| TB |  |  |  |  | 5.399 | 0.020 |
|  | negative | 37 | 26 | 63 |  |  |
|  | positive | 7 | 16 | 23 |  |  |
| AFP |  |  |  |  | 5.957 | 0.015 |
|  | negative | 24 | 12 | 36 |  |  |
|  | positive | 20 | 30 | 50 |  |  |
| GGT |  |  |  |  | 0.745 | 0.388 |
|  | negative | 20 | 23 | 43 |  |  |
|  | positive | 24 | 19 | 43 |  |  |
| PDL1 |  |  |  |  | 4.981 | 0.026 |
|  | negative | 26 | 15 | 41 |  |  |
|  | positive | 15 | 24 | 39 |  |  |
| CTLA4 |  |  |  |  | 2.764 | 0.096 |
|  | negative | 30 | 21 | 51 |  |  |
|  | positive | 11 | 17 | 28 |  |  |
