# Supplementary material for: Uncovering the Association Between m5C Regulator-Mediated Methylation Modification Patterns and Tumour Microenvironment Infiltration Characteristics in Hepatocellular Carcinoma
Source: Front Cell Dev Biol. 2021 Sep 13;9:727935. doi: 10.3389/fcell.2021.727935 (PMC8475949; doi:10.3389/fcell.2021.727935)
Supplement: Supplementary Table 5 — Univariate and multivariate analyses of the factors correlated with the OS of HCC patients. [file Table_5.docx]

**Supplementary Table 5∣**Univariate and multivariate analyses of the factors correlated with the OS of HCC patients.

| **Variables** | **Univariate analysis** | | |  | **Multivariate analysis** | | |
| --- | --- | --- | --- | --- | --- | --- | --- |
|  | **HR** | **95%CI** | **P-value** |  | **HR** | **95%CI** | **P-value** |
| Expression | 2.055 | 1.114-3.790 | 0.021 |  | 2.032 | 1.102-3.748 | 0.023 |
| Sex | 2.730 | 0.660-11.292 | 0.166 |  |  |  |  |
| Grade | 1.751 | 0.954-3.213 | 0.071 |  |  |  |  |
| Age | 1.372 | 0.744-2.531 | 0.311 |  |  |  |  |
| Size | 1.598 | 0.858-2.976 | 0.140 |  |  |  |  |
| T stage | 1.864 | 0.999-3.479 | 0.050 |  | 1.835 | 0.984-3.422 | 0.056 |
| TNM stage | 1.864 | 0.999-3.479 | 0.050 |  |  |  |  |
| cirrhosis | 1.347 | 0.481-3.774 | 0.571 |  |  |  |  |
| HBsAg | 1.086 | 0.520-2.268 | 0.826 |  |  |  |  |
| HBcAb | 0.851 | 0.303-2.387 | 0.759 |  |  |  |  |
| AntiHCV | 0.048 | 0.000-931.063 | 0.547 |  |  |  |  |
| TB | 1.339 | 0.707-2.536 | 0.371 |  |  |  |  |
| ALT | 0.726 | 0.259-2.035 | 0.542 |  |  |  |  |
| ALB | 0.520 | 0.071-3.788 | 0.519 |  |  |  |  |
| AFP | 1.525 | 0.814-2.858 | 0.188 |  |  |  |  |
| GGT | 1.325 | 0.727-2.415 | 0.359 |  |  |  |  |
| PDL1 | 0.881 | 0.476-1.629 | 0.685 |  |  |  |  |
| CTLA4 | 0.601 | 0.291-1.245 | 0.171 |  |  |  |  |
